# Effect of 25 hydroxyvitamin D on attention deficit and hyperactivity in school-age children with ADHD

**DOI:** 10.1097/MD.0000000000035728

**Published:** 2023-10-27

**Authors:** Juan Yang, Huozhong Yuan, Ruijuan Qiu, Xiaoqin Fu

**Affiliations:** a Department of Rehabilitation Medicine, Ganzhou People’s Hospital, Ganzhou, China; b Department of Breast Surgery, Ganzhou People’s Hospital, Ganzhou, China.

**Keywords:** 25 hydroxyvitamin D, attention deficit hyperactivity disorder, level, school age

## Abstract

**Background:**

To observe the serum levels of 25 hydroxyvitamin D [25 (OH) D] in healthy school-age children and children with attention deficit hyperactivity disorder (ADHD) and to analyze the effects of serum 25 (OH) D on the symptoms of attention deficit and hyperactivity in school-age children with ADHD.

**Methods:**

According to the Diagnostic and Statistical Manual of Mental Disorders DSM-IV diagnostic criteria for ADHD in children, 80 healthy children aged 6 years or less than 10 years old and children diagnosed with ADHD in the Department of Rehabilitation Medicine, Department of Pediatrics and Department of Physical Examination of our hospital were randomly selected as research subjects. The serum 25 (OH) D level, attention deficit hyperactivity (Swanson, Nolan, and Pelham, version IV [SNAP-IV] parental version) score and Conners child behavior (PSQ) index were observed and compared between the 2 groups. In addition, the children with ADHD whose serum 25 (OH) D was lower than normal were treated with supplemental VitD3, and the changes in serum 25 (OH) D, SNAP-IV parental score and PSQ index of ADHD children were observed and compared.

**Results:**

Serum 25(OH)D was insufficient or deficient in 26 healthy children, but the SNAP-IV score and PSQ index were normal. Serum 25(OH)D was lower than normal in 69 patients in the ADHD group, which was negatively correlated with SNAP-IV score (*r* = −0.3479, *P* = .0034) and negatively correlated with PSQ index (*r* = −0.3566, *P* = .0026). After vitamin D3 (VitD3) supplementation in 69 children with serum 25(OH)D levels lower than the normal ADHD group, it was found that the SNAP-IV score (*r* = −0.4654, *P* = .0037) and PSQ index (*r* = −0.5680, *P* = .0002) of 34 children with ADHD were negatively correlated with the increase in serum 25(OH)D. The SNAP-IV score and PSQ index of the other 35 children with ADHD showed no correlation with the increase in serum 25 (OH) D (*P* > .05).

**Conclusion subsections:**

Serum 25(OH)D levels lower than normal are more common in school-age children, and levels lower than normal are not the key pathogenic factor of ADHD in school-age children, but serum 25(OH)D levels lower than normal may be the upregulation factor of attention deficit and hyperactivity disorder expression in some school-age children with ADHD. The lower level of serum 25(OH)D may be closely related to the severity of ADHD symptoms in some children.

## 1. Introduction

Attention deficit hyperactivity disorder (ADHD), a persistent neurodevelopmental disorder, has become an important public health problem. ADHD in childhood manifests as serious age-inappropriate impulsivity, hyperactivity and inattention.^[1]^ According to statistics, the prevalence rate of ADHD in school-age children is 3–10%, 2–18% of children worldwide are affected,^[2,3]^ and 30–50% of children will have symptoms lasting to adulthood and the rest of their lives.^[4]^ ADHD in school-age children seriously affects their health, study and life, leading to family and social disharmony. Therefore, an in-depth understanding of the pathogenesis of ADHD is particularly critical for the treatment of ADHD in school-age children.

The exact etiology of ADHD remains unclear. Polygene mutation, micronutrient imbalance, intestinal flora disorder, serotonin (5-HT) and catecholaminergic signaling pathway abnormalities have been confirmed to be related to the onset of ADHD,^[5–8]^ but a unified pathogenesis theory has not yet been formed; therefore, it is particularly critical to study the mechanism of ADHD as a whole and as a system. At present, domestic and foreign scholars have used drugs (methylphenidate, tomoxetine, etc.) and comprehensive rehabilitation treatment (e.g., biofeedback, behavior correction, etc.) for children with ADHD, with limited efficacy.^[9,10]^ Especially for school-age children with ADHD, the above drug treatment easily affects the growth and development of children, and the general acceptance of parents is low. Behavioral therapy and rehabilitation training courses are longer, and children and parents have difficulty adhering to them. Therefore, it is urgent to find a treatment that is easy for children and parents to accept and adhere to.

Dietary nutrition has been increasingly recognized in psychological and behavioral health. We can obtain vitamins, minerals, amino acids, polyunsaturated fatty acids and other nutrients from the diet. However, partial and picky eating is common among school-age children, which directly leads to micronutrient imbalance and affects the internal environment of the body. Studies have shown that the imbalance of vitamin D (VitD) is related to ADHD,^[6]^ and VitD supplementation is also used to treat mental disorders.^[11]^ However, is serum 25 hydroxyvitamin D [25 (OH) D] lower than normal level a pathogenic factor for ADHD of school age? Moreover, the correlation between the lower level of serum 25(OH)D and the severity of attention deficit and hyperactivity in children needs further study.

Based on the above reasons, we observed the serum 25(OH)D levels in healthy school-age children and ADHD children, analyzed the influence of serum 25(OH)D levels lower than normal on hyperactivity and attention deficits in ADHD children, and analyzed the effects of vitamin D3 supplementation on 25-hydroxyvitamin D levels and hyperactivity symptoms.

## 2. Study presentation

### 2.1. Study design

According to the diagnostic criteria for ADHD in children in the Diagnostic Statistical Manual of Mental Disorders DSM-IV,^[12]^ 80 healthy children and 80 children diagnosed with ADHD in the Department of Rehabilitation Medicine, Department of Pediatrics and Department of Physical Examination of our hospital from June 2019 to December 2022 were randomly selected as research subjects. The children in both groups were more than or equal to 6 years old and less than or equal to 10 years old. Serum 25 (OH) D levels, attention deficit hyperactivity scores of Swanson, Nolan, and Pelham, version IV scale, Swanson, Nolan, and Pelham, version IV (SNAP-IV) (Parent edition) and Conners parental symptom questionnaire, PSQ (Parent edition) were observed and compared between the 2 groups. In addition, children in the ADHD group whose serum 25 (OH) D was lower than normal were treated with supplemental VitD3, and the changes in serum 25 (OH) D, SNAP-IV parental score and PSQ index of ADHD children were observed and compared. ADHD was diagnosed by specially trained and qualified physicians from the Department of Rehabilitation Medicine or the Department of Pediatrics of our hospital. The SNAP-IV and PSQ were evaluated by qualified rehabilitation therapists for children. This study was approved by the Ethics Committee of Ganzhou People’s Hospital (TY-ZKY2021-003-01).

### 2.2. Participant data

#### 2.2.1. Healthy children group.

*Inclusion criteria*: Did not meet the diagnostic criteria of ADHD in DSM-IV; the children were more than or equal to 6 years old and less than or equal to 10 years old; 70 ≤ WPPSI-IV score ≤ 110; mother tongue is Mandarin Chinese; no supplements of vitamin D and other health products in recent 1 month; and the family members or guardians of the children were informed of this study, agreed and signed the consent form.

*Exclusion criteria*: Attention deficit and/or hyperactive impulse items in the SNAP-IV rating scale (parent version) ≥ 4; and people with physical diseases, neurological and mental disorders now or in the past.

#### 2.2.2. ADHD children.

*Inclusion criteria*: Meeting the diagnostic criteria for ADHD in DSM-IV; the children were more than or equal to 6 years old and less than or equal to 10 years old; 70 scores ≤ WPPSI-IV score ≤ 110; mother tongue is Mandarin Chinese; have not used any ADHD drugs, and have not supplemented any health products such as vitamin D in recent 1 month; and the family members or guardians of the children were informed of this study, agreed and signed the consent form.

*Exclusion criteria*: Conduct disorder, mood disorder and psychosis; mental retardation, pervasive developmental disorder; obvious physical and neurological diseases; and coagulation dysfunction using any central stimulants, tomoxetine, antipsychotics, or antidepressants.

### 2.3. Assessments

#### 2.3.1. Intelligence assessment.

Using the Wechsler Intelligence Scale for Children, Version V,^[13]^ 2 specially trained therapists from the Department of Rehabilitation Medicine who have obtained the intelligence assessment qualification of Wechsler will conduct the intelligence quotient (IQ) test according to the standard methods stipulated in the test manual. The scale includes 4 indexes of speech comprehension, perceptual reasoning, working memory and processing speed, among which verbal comprehension and perceptual reasoning are the general ability index and working memory and processing speed are the cognitive efficiency index. The Wechsler Intelligence Scale for Children, Version V has 10 core subtests for building blocks, similarity, number memorization, picture concept, decoding, vocabulary, letters, numbers, matrix reasoning, comprehension and coincidence retrieval. The higher the score, the higher the intelligence. According to the WSC-V measure, IQ ≥ 120, 110-119, 90-109, 80-89, and 70-80 were classified as excellent, upper middle, medium, lower middle, and critical, respectively. Children with IQ scores less than 70 and greater than 110 were excluded from the study.

#### 2.3.2. Parent Symptom Questionnaire (PSQ) Index of Conners.^[14]^

Professionals guide parents to complete the Conners Parent Assessment Questionnaire (PSQ). The scale includes 48 items in 5 subscales (conduct problems, learning problems, psychosomatic problems, impulsive hyperactivity, and anxiety). A four-level scoring method (0,1,2,3) was adopted. 0 score: no such problem; 1. Occasionally a little or slightly present; 2 points: frequent or more serious; 3 points: very common or very serious. The score of the project was added and divided by the number of items to obtain the hyperactivity index. A hyperactivity index of 0.75 to 1.5 indicates mild hyperactivity, 1.5 to 2.25 indicates obvious hyperactivity, and a hyperactivity index ≥ 2.25 indicates severe hyperactivity.

#### 2.3.3. ADHD Score.

The Chinese version of Swanson, Nolan, and Pelham, Version IV scale, SNAP-IV (Parent edition) revised by Zhou Jinbo was adopted,^[15]^ which included 3 subscales and 26 items. They were divided into the attention inattention scale (items 1–9), hyperactivity/impulsivity scale (items 10–18), and oppositional disobedience scale (items 19–26). Each item was scored according to 4 levels from 0 to 3: 0 points: none; 1 point: a little bit; 2 points: not a lot; and 3 points: a lot. The score value is calculated by adding the scores of each item and dividing by the number of items. A score of 0–1 is normal; a score of 1.1–1.5 hints at an edge; a score of 1.6–2 indicates moderate; and a score of 2 or above indicates serious. The higher the score, the more severe the ADHD symptoms.

### 2.4. Blood parameters

25(OH)D detection^[16]^: 2 mL of fasting venous blood was collected in the morning, and the serum was isolated and stored at −70°C. Chemiluminescence immunoassay was used to detect the level of 25(OH)D in the serum, which was divided into 3 horizontal segments: adequate level (20–100 ng/mL); insufficient level (15–19.9 ng/mL); and deficient level (<15 ng/mL). We defined < 20 ng/mL as below normal.

### 2.5. Data analysis

The SPSS 15.0 statistical software package was used for data analysis. Measurement data conforming to a normal distribution were expressed as $\bar{x}\pm s$, and a group *t* test was used for comparison. The χ^2^ test was used for enumeration data, and Pearson correlation analysis was used for correlation analysis. *P* < .05 was considered statistically significant.

## 3. Results

### 3.1. General information about the subjects

The gender, age and IQ scores of the subjects in this study showed no statistical significance (*P* > .05), indicating comparability (Table 1).

**Table 1:** General information of the subjects (n).

Group	n	Gender (male/female)	Age (yr, $\bar{x}\pm s$)	IQ score (points, $\bar{x}\pm s$)
Healthy children group	80	52/28	7.32 ± 2.16	87.56 ± 12.35
ADHD children group	80	54/26	7.46 ± 2.08	84.24 ± 13.53
χ^2^/*t* value	–	0.112*	0.418†	−1.621†
*P* value	–	.738	.677	.107

IQ = intelligence quotient.

*χ ^2^ value.

† *t* value.

### 3.2. Comparison of indicators between the 2 groups

In the healthy children group, serum 25(OH)D was lower than the normal level in 26 cases, but the SNAP-IV score and PSQ index were normal. The other 54 children had normal levels of serum 25(OH)D. The serum 25(OH)D level, SNAP-IV score and PSQ index of the healthy children group were not statistically significant (*P* > .05) (Table 2).

**Table 2:** Comparison of indicators in the healthy children group ($\bar{x}\pm s$).

Indicator/group	Healthy children group (n = 80)	
	Group with normal serum 25 (OH) D level (n = 54)	Serum 25 (OH) D was lower than normal group (n = 26)
Serum 25 (OH) D levels (ng/mL)	35.12 ± 14.38	16.58 ± 3.24
SNAP-IV (Parent Edition) score	0.43 ± 0.55	0.41 ± 0.52
* P* value	.278*	.196*
PSQ index	0.62 ± 0.08	0.56 ± 0.14
* P* value	.463*	.352*

25 (OH) D = 25 hydroxyvitamin D.

* No statistical significance and showed no correlation.

**Figure 1.**
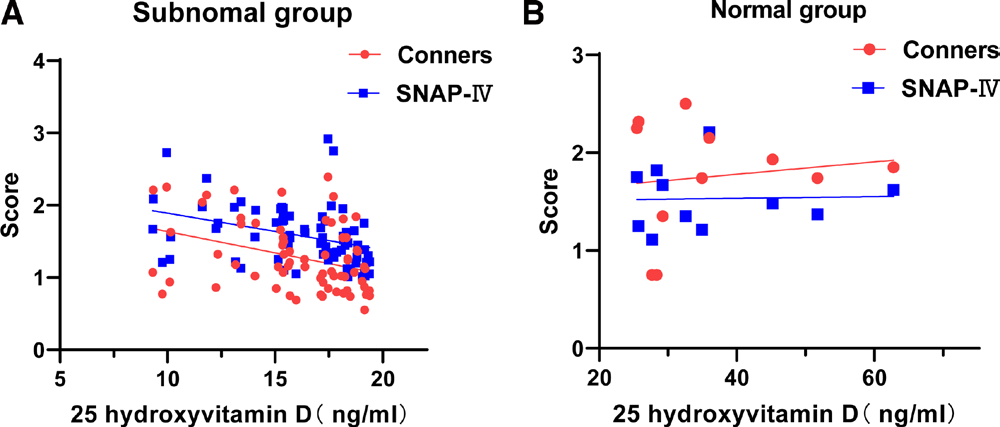
(A) The serum 25(OH)D level in 69 patients in the ADHD group was lower than the normal level, and the degree of lower than the normal level was negatively correlated with the SNAP-IV score and PSQ index (*P* < .01). (b) The serum 25(OH)D level in the remaining 11 patients was at an adequate level, and there was no correlation with the SNAP-IV score or PSQ index (*P* > .05). 25 (OH) D = 25 hydroxyvitamin D, ADHD = attention-deficit hyperactivity disorder, SNAP-IV = Swanson, Nolan, and Pelham, version IV.

The serum 25(OH)D level in 69 patients in the ADHD group was lower than the normal level, and a level lower than the normal level was negatively correlated with the SNAP-IV score and PSQ index (*P* < .01). The serum 25(OH)D level in the remaining 11 patients was at an adequate level, and there was no correlation with the SNAP-IV score and PSQ index (*P* > .05) (Table 3, Fig. 1A and B).

**Table 3:** Correlation analysis of serum 25(OH)D level with SNAP-IV score and PSQ index in the ADHD group ($\bar{x}\pm s$).

Indicator/group	ADHD children group (n = 80)	
	Group with normal serum 25 (OH) D level (n = 11)	Serum 25 (OH) D was lower than normal group (n = 69)
Serum 25 (OH) D levels (ng/mL)	36.33 ± 12.04	16.11 ± 2.832
SNAP-IV (Parent Edition) score	1.531 ± 0.3225	1.582 ± 0.4109
* r* value	0.03298	−0.3479
* P* value	.9233	.0034
PSQ指数	1.76 ± 0.59	1.276 ± 0.4678
* r* value	0.1286	−0.3566
* P* value	.7062	.0026

25 (OH) D = 25 hydroxyvitamin D, ADHD = attention-deficit hyperactivity disorder, SNAP-IV = Swanson, Nolan, and Pelham, version IV.

**Figure 2.**
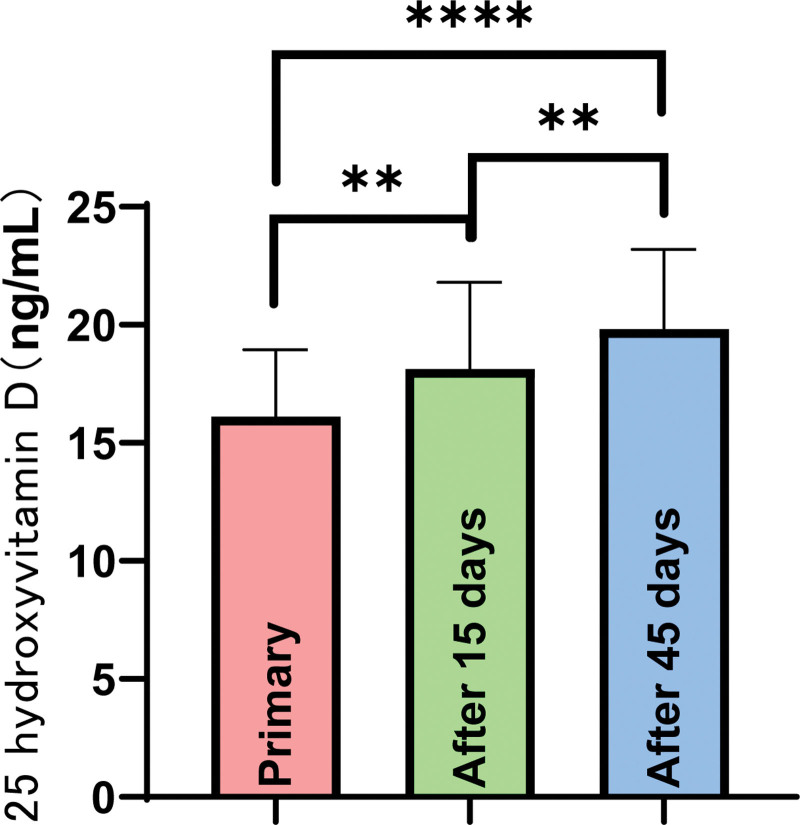
There were significant differences before and after 15 days of supplementation and after 15 and 45 days of supplementation (*P* < .01), and the difference was more significant before and 45 days after supplementation (*P* < .0001). ** *P* < .01; **** *P* < .0001.

Sixty-nine children with ADHD whose serum 25(OH)D levels were lower than normal were treated with VitD3 supplementation (daily VitD31000IU), and serum 25(OH)D levels were observed before supplementation and 15 and 45 days after supplementation. There were significant differences in serum 25(OH)D levels at 3 stages by one-way analysis of variance. There were significant differences before and after 15 days of supplementation and after 15 and 45 days of supplementation (*P* < .01), and the difference was more significant before and 45 days after supplementation (*P* < .0001). These results indicated that the intervention of VitD3 supplementation could improve the symptoms of attention deficit and hyperactivity in some school-age ADHD children with serum 25 (OH) D lower than the normal level, and the therapeutic effect tended to be more significant with the extension of treatment time (Table 4, Fig. 2).

**Table 4:** Comparison of serum 25(OH)D levels before and after supplementation with VitD_3_ in the ADHD group ($\bar{x}\pm s$).

Serum 25 (OH) D levels (ng/mL)	n	Mean ± SD	*F*	*P*
At first visit	69	16.11 ± 2.832	21.84	<.0001
After 15 d of treatment	69	18.14 ± 3.652	–	–
After 45 d of treatment	69	19.82 ± 3.379	–	–
At first visit vs After 15 d of treatment	–	–	–	.0011
At first visit vs After 45 d of treatment	–	–	–	<.0001
After 15 d of treatment vs After 45 d of treatment	–	–	–	.0087

25 (OH) D = 25 hydroxyvitamin D, ADHD = attention-deficit hyperactivity disorder.

**Figure 3.**
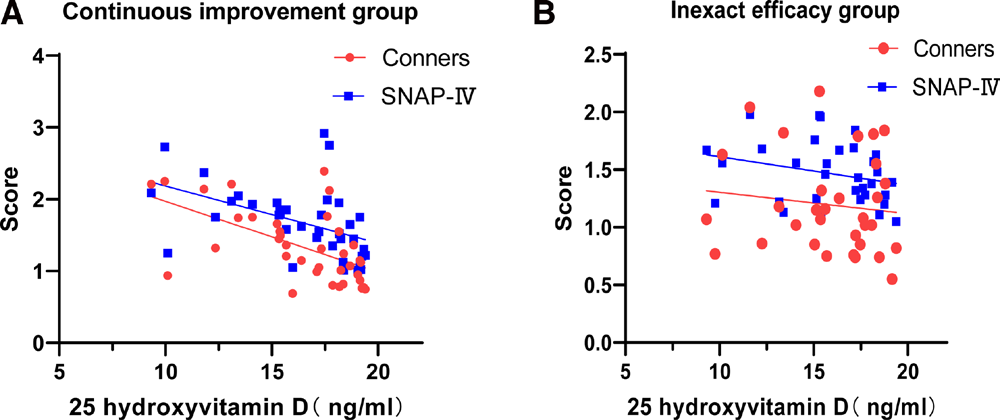
(A) Through VitD3 supplementation, the SNAP-IV score and PSQ index were negatively correlated with the increase in serum 25 (OH) D in 34 children with ADHD (*P* < .01). (b) Through VitD3 supplementation, the SNAP-IV score and PSQ index of the other 35 ADHD children showed no correlation with the increase in serum 25 (OH) D (*P* > .05). 25 (OH) D = 25 hydroxyvitamin D, ADHD = attention-deficit hyperactivity disorder, SNAP-IV = Swanson, Nolan, and Pelham, version IV.

Through VitD3 supplementation in 69 children with ADHD whose serum 25 (OH) D was lower than the normal level, we further found that the SNAP-IV score and PSQ index were negatively correlated with the increase in serum 25 (OH) D in 34 children with ADHD (*P* < .01). These 34 cases were included in the continuous improvement group. The SNAP-IV score and PSQ index of the other 35 ADHD children showed no correlation with the increase in serum 25 (OH) D (*P* > .05), and we included these 34 patients in the uncertain effect group (Table 5, Fig. 3A and B).

**Table 5:** Comparison of the data between the 2 ADHD groups ($\bar{x}\pm s$).

Indicator/group	Serum 25 (OH) D was lower than normal group (n = 69)	
	Continuous improvement group (n = 34)	Inexact efficacy group (n = 35)
Serum 25 (OH) D levels (ng/mL)	16.34 ± 2.846	15.84 ± 2.835
SNAP-IV (Parent Edition) score	1.681 ± 0.4863	1.468 ± 0.2659
* r* value	−0.4654	−0.2644
* P* value	.0037	.1436
PSQ指数	1.345 ± 0.4945	1.196 ± 0.4286
* r* value	−0.5680	−0.1228
* P* value	.0002	.5030

25 (OH) D = 25 hydroxyvitamin D, ADHD = attention-deficit hyperactivity disorder, SNAP-IV = Swanson, Nolan, and Pelham, version IV.

## 4. Discussion

In a large sample of more than 20,000 cases and 30,000 controls, 12 genome-wide statistically significant loci were identified by the International Psychiatric Genomic Consortium. Related genes include microtubule skeleton components, the cadherin family, transcription factors, and axon-directing genes. Many of them are related to neural development,^[7]^ indicating that most ADHD patients have genetic mutations.

At present, the relationship between an imbalance in neurotransmitter secretion and ADHD is still a research hotspot. Neurotransmitters such as dopamine (DA), norepinephrine (NE) and 5-HT can regulate cognitive functions such as attention, motivation, consciousness, executive function, sensory gating, social behavior and impulsiveness.^[17,18]^ Changes in nutrients in the dietary environment may affect the composition and metabolism of intestinal flora, suggesting that dietary intervention can be used to adjust the distribution of intestinal flora and stimulate the beneficial function of intestinal flora,^[19]^ and different strains of intestinal flora can regulate a variety of monoamine neurotransmitters, including 5-HT, catecholamine, and R-aminobutyric acid.^[20]^ Clarke found that 5-HT is regulated by intestinal flora, and 95% of 5-HT in the human body is produced by intestinal epithelial cells.^[21]^ 5-HT is an inhibitory neurotransmitter that plays an important role in the regulation of defense and anxiety-depression emotions.^[22]^ 5-HT receptors are divided into 7 families and 14 subtypes, among which inhibition of the 5-HT1A receptor or 5-HT7 receptor can reduce the level of 5-HT in the body, resulting in hyperactivity and inattention.^[23]^

Experimental studies in mice confirmed that the 5-serotonin transporter-associated promoter region involved in the transcriptional regulation and transport of 5-HT was prone to genetic variation, including insertion and loss of 44 base pairs, forming alleles of 2 different lengths, l (long) and s (short) genes. ADHD individuals with the 5-serotonin transporter-associated promoter region s allele are especially sensitive to environmental changes.^[24,25]^ Kesby found that VitD deficiency during development could lead to disorders of a variety of neurotransmitter systems in the brains of newborn rats, including 5-HT, DA, and NE.^[26]^ In this study, some children in both groups had serum 25(OH)D levels lower than the normal level (11 cases in the healthy children group and 69 cases in the ADHD group), indicating that serum 25(OH)D levels lower than the normal level are not the key pathogenic factor of ADHD in school-age children. Why do only children with ADHD express hyperactivity and attention deficits? In summary, we conclude that insufficient or deficient serum 25(OH)D is not the key pathogenic factor for ADHD in school-age children. It is possible that the body may have an imbalance in neurotransmitter secretion when the diet environment changes on the basis of genetic variation and gene mutation, and the expression of attention deficit and hyperactivity in ADHD children is easily upregulated.

Our research group showed that the degree of serum 25(OH)D lower than the normal level in 69 children with ADHD was negatively correlated with the SNAP-IV score and PSQ index (*P* < .01). After supplementing VitD3 1000 IU/d for these 69 children with ADHD, it was found that after supplementing VitD3 15 and 45 days, in 34 cases, the SNAP-IV score and PSQ index were negatively correlated with the increase in serum 25 (OH) D (*P* < .01), and the symptoms of attention deficit and hyperactivity showed continuous improvement (continuous improvement group). The SNAP-IV score and PSQ index of the other 35 children showed no correlation with the increase in serum 25 (OH) D (*P* > .05), and there was no improvement in the symptoms of attention deficit and hyperactivity (uncertain efficacy group). There were significant differences in serum 25 (OH) D levels in 69 children before VitD supplementation and 15 and 45 days after VitD supplementation (*P* < .01), and the difference was more significant before and 45 days after supplementation (*P* < .0001), indicating that the lower level of serum 25 (OH) D than the normal level may be the upregulating factor for the expression of attention deficit and hyperactivity in some school-age children with ADHD, and the lower level of serum 25 (OH) D than the normal level may be closely related to the severity of attention deficit and hyperactivity in some children. The intervention of VitD3 supplementation can improve the symptoms of attention deficit and hyperactivity in some school-age ADHD children with serum 25 (OH) D lower than the normal level, and the therapeutic effect tends to be more significant with the extension of treatment time.

At the same time, there were also 11 children in the ADHD group whose serum 25 (OH) D was at a sufficient level, showing no correlation with SNAP-IV score and PSQ index (*P* > .05). There was no correlation between the level of serum 25(OH)D deficiency and the severity of ADHD symptoms in 35 children with ADHD (*P* > .05). What is the reason? Domestic and foreign research results on micronutrients other than VitD suggest that the imbalance of vitamin A (VitA), VitD, vitamin B2 (VitB2), vitamin B6 (VitB6), vitamin B12 (VitB12), folic acid, iron, zinc, magnesium, copper and other micronutrients are also correlated with ADHD,^[27,28]^ and DA is the most active neurotransmitter among catecholamines. Three dopaminergic pathways in the brain have been proven to be related to the pathogenesis of ADHD: mesolimbic, mesocortal and substantia nigrostriatum pathways.^[29]^ Therefore, we speculate that school-age children with ADHD may have different genetic variations, gene mutations or different neurotransmitter pathway disorders (such as DA pathway, 5-HT pathway, etc.). When picky eating leads to the deficiency of micronutrients (VitA, VitB2, VitB6, VitB12, VitD, iron, magnesium, zinc, copper, etc. It may affect different pathways and lead to insufficient secretion of neurotransmitters, which may upregulate the expression of hyperactivity and attention deficits in children. These school-age children with ADHD should pay attention to the neurotransmitter pathways affected by genetic variation, gene mutation and other factors, find the micronutrients that promote the secretion of this neurotransmitter, and regulate the expression of hyperactivity and attention deficit symptoms in children by supplementing these micronutrients. This is consistent with foreign scholars’ thinking of mineral supplementation for the treatment of mental disorders.^[28]^

In the next study, we will classify school-age children with ADHD (inattentive type, hyperactive type and mixed type). To further study the different neurotransmitter pathways (DA, NE, etc.) disorders in school-age children with ADHD of different types caused by different genetic variations and gene mutations and downregulation of attention-deficit and hyperactivate-like micronutrients (such as VitA, VitB2, VitB6, VitB12, iron, magnesium, zinc, etc.) in school-age children with ADHD.

## Acknowledgements

We thank the children and their parents for participating in our study.

## Author contributions

**Conceptualization:** Juan Yang.

**Data curation:** Juan Yang, Ruijuan Qiu, Xiaoqin Fu.

**Methodology:** Juan Yang, Ruijuan Qiu, Xiaoqin Fu.

**Project administration:** Huozhong Yuan.

**Writing – original draft:** Juan Yang.

**Writing – review & editing:** Huozhong Yuan.
